# Supplementary material for: Melanopic stimulation does not alter psychophysical threshold sensitivity for luminance flicker
Source: Sci Rep. 2021 Oct 11;11:20167. doi: 10.1038/s41598-021-99684-0 (PMC8505480; doi:10.1038/s41598-021-99684-0)
Supplement: Supplementary file 1 — Supplementary Information. [file 41598_2021_99684_MOESM1_ESM.pdf]

Melanopic stimulation does not alter psychophysical threshold sensitivity for luminance flicker

Authors: Joris Vincent[1*], Edda B. Haggerty[2], David H. Brainard[3], Geoffrey K. Aguirre[2]

1. Computational Psychology, Institute of Software Engineering and Theoretical Computer Science, Technische Universität Berlin, Germany
2. Department of Neurology, Perelman School of Medicine, University of Pennsylvania, Philadelphia PA
3. Department of Psychology, School of Arts and Sciences, University of Pennsylvania, Philadelphia PA

*Corresponding author: Joris Vincent, joris.vincent@tu-berlin.de

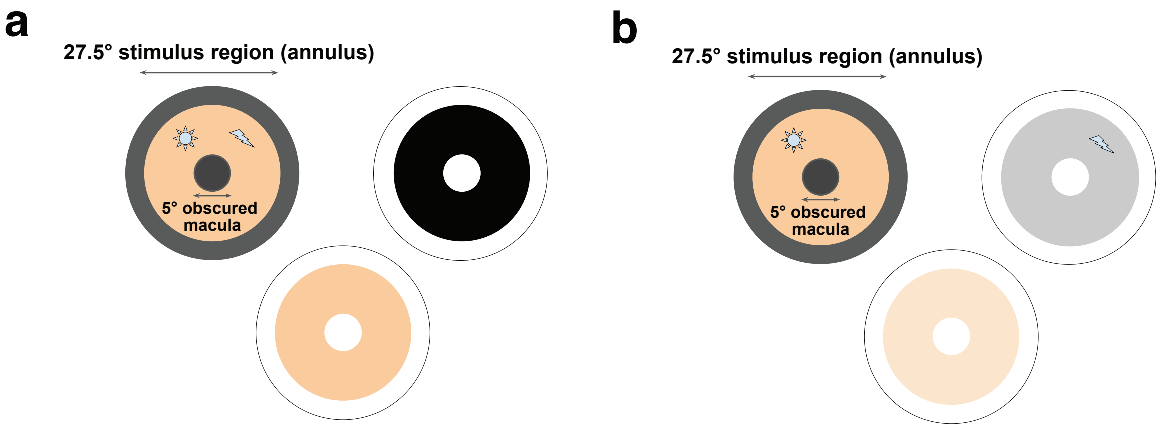

Supplementary Figure S1. Spatial structure of stimulus and stimulation sources. a) Stimulus configuration for Experiments 1 and 2. Both modulation of melanopic stimulation (sun icon) and luminance flicker (lightning bolt icon) were provided by modulation of the spectral output of the OneLight (top left). The combined melanopsin and flicker stimulus subtended 27.5 degrees visual angle (orange field), with the central 5 degree macular region and far periphery extensions optically blocked. To prevent light-scatter on these blocked regions from being visible, steady light from the DLP was added in a circular central region and annular outer region, at max DLP output, with the other DLP pixels set to off (top right). This light was optically admixed with the stimulus from the OneLight, such that to the Participant, this admixed light covered the extent of the blocked regions (bottom). b) Stimulus configuration for Experiment 3, in which the light-flux flicker stimulus (lightning bolt icon) was provided by the DLP (top right). Output from the OneLight (top left) was used only to modulate the melanopic content of the background (orange field). To allow for flicker, the DLP component had to have a background component as well, indicated by the light gray field (top right). To the participant, this meant that in the absence of flicker, the stimulus region of the combined stimulus looked brighter than in Experiments 1 & 2 (lighter orange field, bottom). In Experiment 3, the DLP was also used to add steady light to the central and outer blocked regions of the stimulus, as in Experiments 1 and 2.

Supplementary Figure S2:

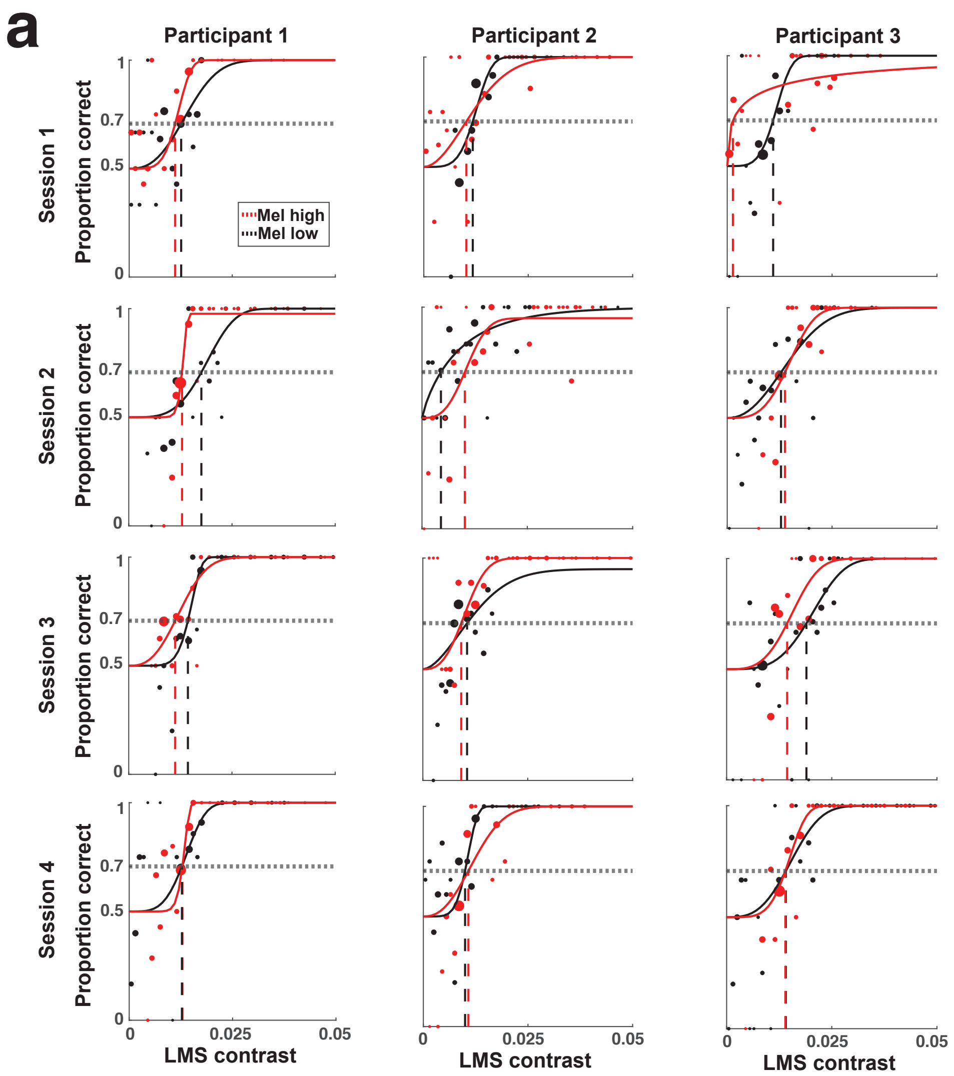

Supplementary Figure S2. Psychometric functions for each session, participant, and experiment. a) Experiment 1; columns for participants 1-3, and rows for sessions 1-4. Dots indicate proportion correct for each LMS contrast level, specified on the x-axis directly and not in percent, with 0.05 indicating 5% contrast. Trials are binned per 0.001 contrast, with dot size indicating the number of trials in each bin. Black: low melanopsin condition; Red: high melanopsin condition. Curves indicate best fitting Weibull function to each condition. Dotted horizontal line indicates the criterion for threshold readout (0.7071); colored dotted vertical lines indicate the threshold readout for each condition. b) Experiment 2; columns for participant 4-6. Note that in one case (Participant 5, Session 1, high melanopsin condition), the estimated threshold lies outside of the plotted x-axis range. c) Experiment 3; columns for participants 3, 5, 6; and rows for sessions 1-6. Proportion correct is shown for change in DLP RGB value (delta RGB), binned per DLP RGB unit. (Figure continued on next two pages).

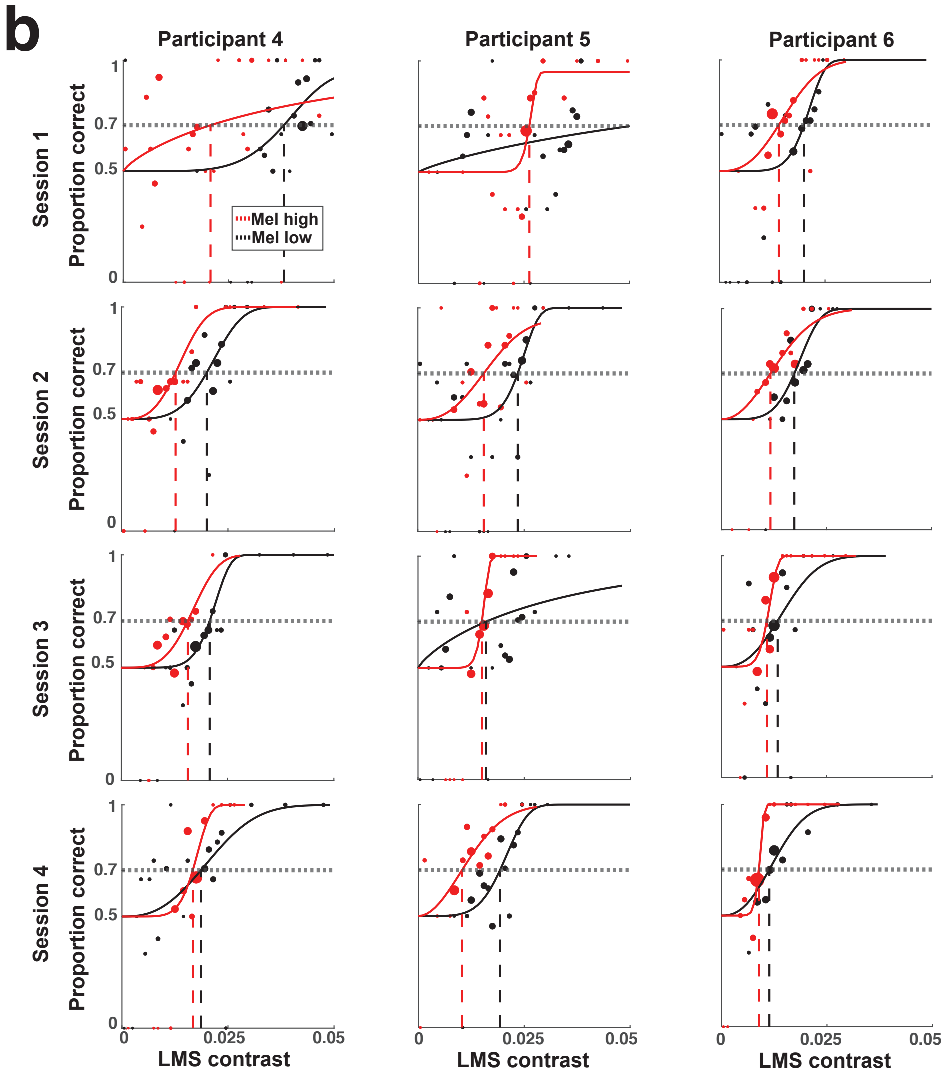
b
Participant 4
Participant 5
Participant 6
Session 1
Session 2
Session 3
Session 4
Proportion correct
Mel high
Mel low
1
0.7
0.5
0
0.025
0.05
LMS contrast

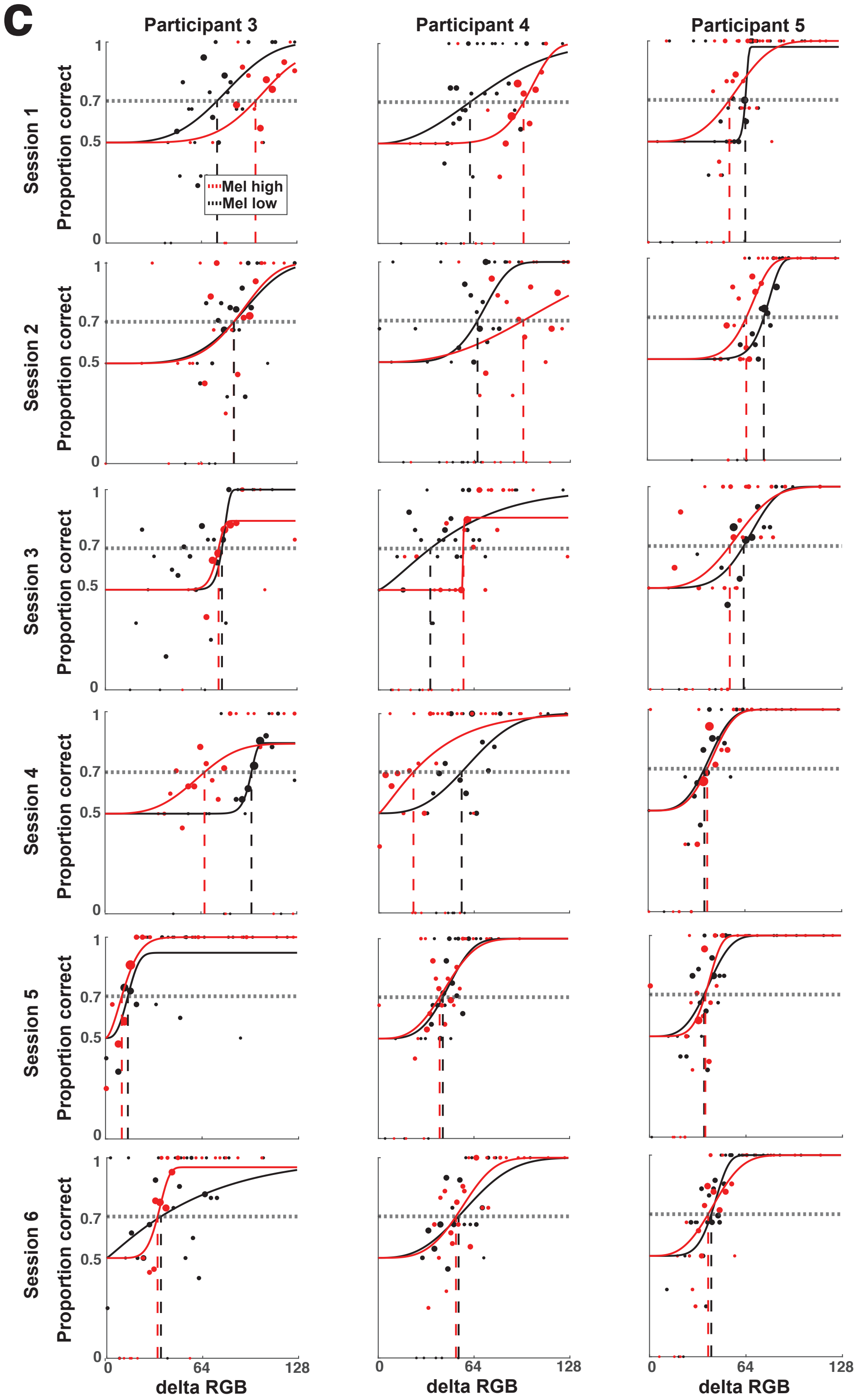
c
Participant 3
Participant 4
Participant 5
Session 1
Session 2
Session 3
Session 4
Session 5
Session 6
Proportion correct
Mel high
Mel low
0
0.5
0.7
1
0
64
128
delta RGB

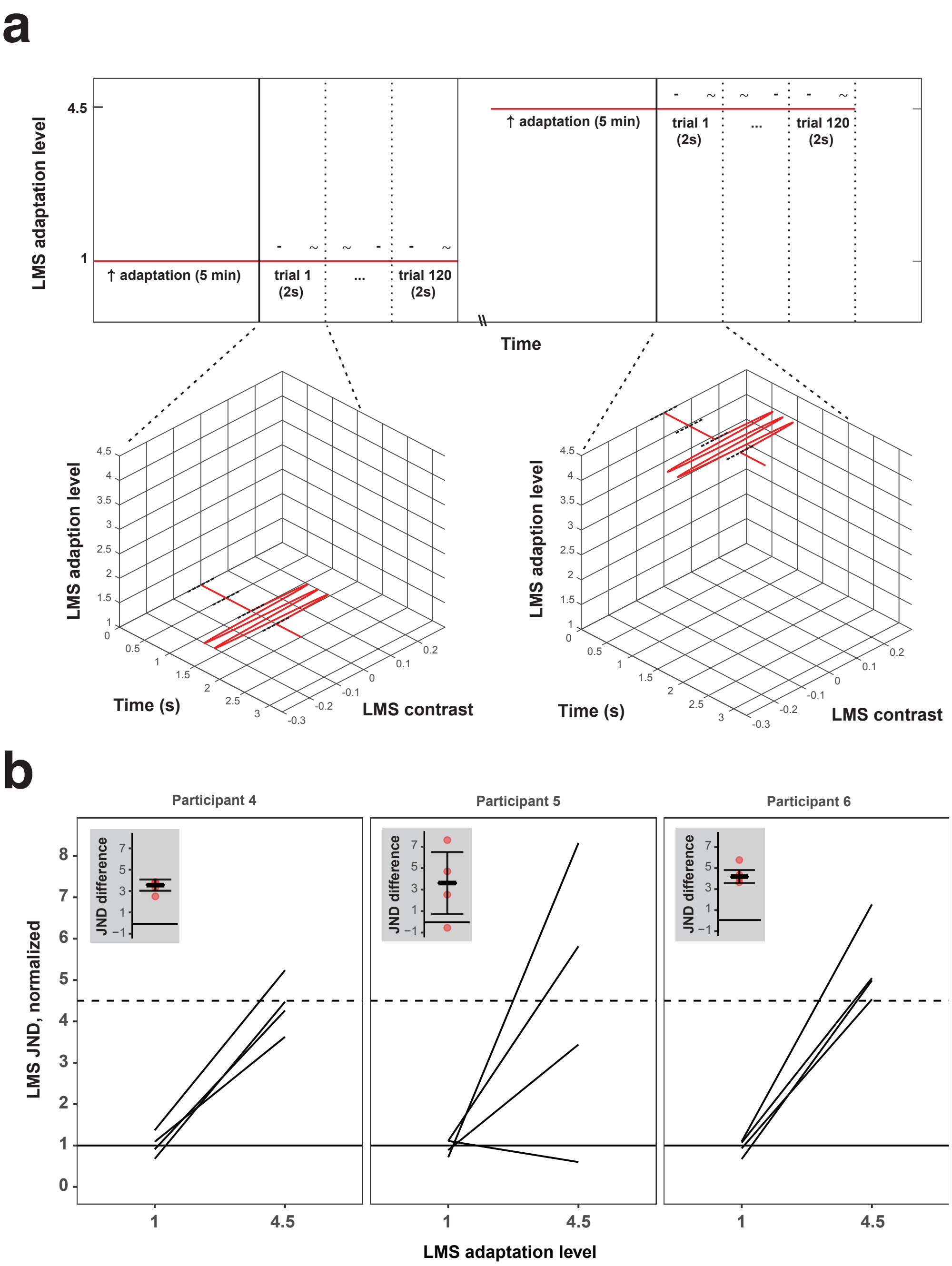

Supplementary Figure S3. Experiment 1 positive control stimulus and results. **a)** Each block contained only a single level of L+M+S-cone directed stimulation (vertical axis gives normalized luminance: 1 or 4.5) which remained constant through the entirety of that block. Blocks started with an adaptation period of 5 minutes, during which the participant fixated on the background for that block. Participants then completed 120 trials of a 2IFC task. Individual trials consisted of two 500 ms intervals, each followed by a 500 ms ISI. During either the first or second interval, sinusoidal flicker (5 Hz) directed at the L, M, and S cones was presented around the background (low-LMS background case shown on left, high-LMS background case shown on right); during the other interval no such flicker was presented. After both intervals were presented, participants were asked to indicate which of the two intervals contained the flicker – this response was untimed. Intervals were indicated with an auditory cue. Participants could take a variable-length break in between blocks. Block-order was pseudorandom. **b)** Control results. Normalized cone-directed flicker detection thresholds on low- and high-LMS backgrounds, for the three participants in Experiment 1. Dark gray lines express thresholds as Just-Noticeable-Difference (JND) from the background on four single sessions. Solid blue lines indicate the median JNDs for each participant across four sessions. All JNDs for a participant were normalized to that participant's median JND on the low-melanopic background. Error bars indicate +/- 1 standard error of the median. If the cone-directed flicker detection is mediated by background LMS stimulation in a Weber's law fashion, JNDs on the 4.5x higher LMS background should be 4.5x higher (difference of 3.5 in the normalized JND representation) than on the low-LMS background (dashed horizontal line). Insets: The four red dots show the within-session differences between JNDs at low- and high-LMS adaptation level. The thicker horizontal line and gray whiskers show the median and 95% confidence interval of the four within-session differences.
